# Vaccatides: Antifungal Glutamine-Rich Hevein-Like Peptides from *Vaccaria hispanica*

**DOI:** 10.3389/fpls.2017.01100

**Published:** 2017-06-21

**Authors:** Ka H. Wong, Wei Liang Tan, Shruthi G. Kini, Tianshu Xiao, Aida Serra, Sui Kwan Sze, James P. Tam

**Affiliations:** School of Biological Sciences, Nanyang Technological UniversitySingapore, Singapore

**Keywords:** *Vaccaria hispanica*, hevein-like peptides, anti-fungal, cysteine-rich peptides, hevein

## Abstract

Hevein and hevein-like peptides are disulfide-constrained chitin-binding cysteine-rich peptides. They are divided into three subfamilies, 6C-, 8C-, and 10C-hevein-like peptides, based on the number of cysteine residues. In addition, hevein-like peptides can exist in two forms, short and long. The long C-terminal form found in hevein and 10C-hevein-like peptides contain a C-terminal protein cargo. In contrast, the short form without a protein cargo is found in all three subfamilies. Here, we report the discovery and characterization of two novel glutamine-rich and protein cargo-free 8C-hevein-like peptides, vaccatides vH1 and vH2, from *Vaccaria hispanica* of the Caryophyllaceae family. Proteomic analyses showed that the vaccatides are 40–41 amino acids in length and contain a chitin-binding domain. NMR determination revealed that vaccatide vH2 displays a highly compact structure with a N-terminal cystine knot and an addition C-terminal disulfide bond. Stability studies showed that this compact structure renders vaccatide vH2 resistant to thermal, chemical and proteolytic degradation. The chitin-binding vH2 was shown to inhibit the mycelium growth of four phyto-pathogenic fungal strains with IC_50_ values in the micromolar range. Our findings show that vaccatides represent a new family of 8C-hevein-like peptides, which are protein cargo-free and glutamine-rich, characteristics that differentiate them from the prototypic hevein and the 10C-hevein-like peptides. In summary, this study enriches the existing library of hevein-like peptides and provides insight into their molecular diversity in sequence, structure and biosynthesis. Additionally, their highly disulfide-constrained structure could be used as a scaffold for developing metabolically and orally active peptidyl therapeutics.

## Introduction

*Vaccaria hispanica*, belonging to the Caryophyllaceae family, is an herbaceous flowering plant native to China. Dried *V. hispanica* seeds, also known as cowherb seeds or Wang Bu Liu Xing (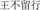), have been widely used as a traditional Chinese medicine to promote lactation, stimulate blood circulation, regulate menstruation, promote urination and relieve inflammation(Efthimiadou et al., 2012). Major chemical compounds reported in *V. hispanica* seeds are metabolites, which include triterpenoid saponins, polyphenols, flavonoids, xanthones, and alkaloids (Zhou et al., 2016). Recently, eight orbitides, which are cyclic peptides with five to eight aliphatic aa, were characterized and shown to display cytotoxic, anthelmintic and estrogen-like activities (Itokawa et al., 1995).

Our interest in the discovery and design of orally active peptides has led us to undertake a program focusing on CRPs in medicinal plants (Tam et al., 1999, 2015; Nguyen et al., 2011a,b, 2012, 2013, 2014, 2015; Kini et al., 2015; Kumari et al., 2015). Plant CRPs, particularly those containing six to ten cysteine residues with molecular weights ranging from 2 to 6 kDa, not only have large footprints required for high pharmacological specificity and selectivity, but are highly disulfide-constrained and structurally compact, properties that provide them high metabolic stability and the potential as orally active compounds (Nguyen et al., 2011a,b, 2012, 2013).

In our discovery program of CRPs in medicinal plants, the most frequently encountered family belongs to the carbohydrate-binding modular peptides, hevein-like peptides. These peptides, consisting 29 to 45 aa in length, are an under-explored family of CRPs (Tam et al., 2015). In 1960, hevein, the first member of 8C-hevein-like peptide, was isolated from the *Hevea brasiliensis* latex (Archer, 1960). Since its discovery nearly 60 years ago, only about 40 hevein-like peptides have been characterized from 14 different plant species in ten different families, including Amaranthaceae, Ginkgoaceae, Poaceae, and Moringaceae (Broekaert et al., 1992; Nielsen et al., 1997; Lipkin et al., 2005; Odintsova et al., 2009; Wong et al., 2016).

Hevein-like peptides are distinguished from other CRPs by the presence of a highly conserved chitin-binding motif. Chitin, the second most abundant polymer after cellulose, is a β-1,4-*N*-acetyl-D-glucosamine polymer found in the fungal cell walls as well as the insects and arthropods exoskeletons (Younes and Rinaudo, 2015). The ability to bind to chitin together with the presence of specific decorated groups of aa enable hevein-like peptides to play a role in host defense, such as antifungal activity in plants (Koo et al., 1998; Wong et al., 2016).

**FIGURE 1:**
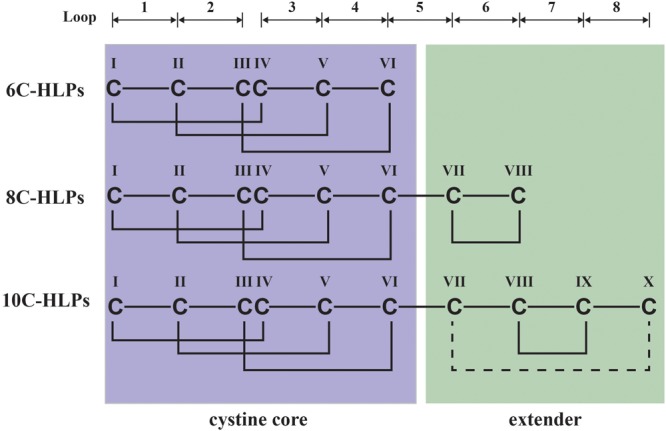
Schematic diagram of hevein-like peptides (HLPs) subfamilies. The backbone segments between cysteine residues are divided into loops and labeled as 1–8. Hevein-like peptides are classified into three sub-families: 6C-, 8C-, and 10C-hevein-like peptides. 8C-Hevein-like peptide are the prototypic member of this family, whereas 6C-hevein-like peptide is the truncation version with the disulfide bond at the extender was removed. 10C-Hevein-like peptide have an additional disulfide bond between C-terminal and loop 5.

Hevein and hevein-like peptides share a common cystine-knot core arrangement, but they display a wide range of cysteine motifs, disulfide connectivity, and precursor arrangements unmatched in plant CRPs. In general, they are divided into three subfamilies, 6C-, 8C-, and 10C-hevein-like peptides, based on the number of cysteine residues present, with each subfamily containing different numbers of intercysteinyl loops (**Figure 1**). The 8C-hevein-like peptides can be regarded as prototypes because of their bipartite arrangement. At their N-terminus is a cystine-knot core consisting of approximately 30 residues, three disulfide pairs and a chitin-binding domain in loops 3 and 4. At their C-terminus is an extender fragment comprised of >12 residues and two additional cysteine residues forming the fourth disulfide bond (Tam et al., 2015). The cystine-knot core is present in all hevein-like peptides, but the extender is absent in the 6C-hevein-like peptides, which are truncated 8C-hevein-like peptides (Kini et al., 2015). On contrary, the fifth disulfide bond in 10C-hevein-like peptides is located either within the C-terminus extender or connected between the extender and the cystine-knot core. To exemplify, the fifth disulfide bond is inter-connected between the extender and loop 5 in the Ee-CBP from spindle tree *Euonymus europaeus* (Van den Bergh et al., 2002), and in *Eucommia ulmoides* EAFP, it is located between loops 1 and 7 (Huang et al., 2002).

Hevein-like peptides are also classified under a broad scheme as carbohydrate-binding modules which include lectins. As such the modular hevein-like peptides can be found as dimer or tetramer in UDA from *Urtica dioica* (Beintema and Peumans, 1992) and WGA from *Triticum aestivum* (Wright, 1992), respectively.

Biosynthetically, the precursors of hevein and hevein-like peptides share a three-domain arrangement comprising a signal peptide domain, a mature hevein-like peptide domain and a C-terminal domain. However, the lengths of the C-terminal domains are highly variable ranging from 13 to 254 residues. Again, they can be broadly classified based on their biosynthetic precursors into two classes: long and short. The long C-terminal domains contain a hinge region followed by a protein cargo such as a class I chitinase-like (Van den Bergh et al., 2004) or barwin-like domain (Broekaert et al., 1990). In contrast, the short C-terminal domains consist of a remnant peptide without a protein cargo. In addition, hevein-like peptides can also be rich in certain aa other than cysteine. For example, most hevein-like peptides are glycine-rich. Recently, our laboratory has characterized a proline-rich subfamily of 8C-hevein-like peptides, ginkgotides from the Ginkgoaceae family of gymnosperm (Wong et al., 2016). The presence of proline flanking both the N-terminus and C-terminus gives the ginkgotides extraordinarily high resistance against degradation by exopeptidases, an added advantage to the known stability of the disulfide-constrained peptides (Nguyen et al., 2011a, 2015; Kumari et al., 2015; Wong et al., 2016). Such stabilities of hevein-like peptides and other plant CRPs qualify them as potential active components in herbal medicines, that normally administrated as decoctions, infusions or teas (Wong et al., 2011; Tam et al., 2015).

Here, we report the discovery and characterization of a novel glutamine-rich subfamily in the 8C-hevein-like peptides, vaccatides vH1 and vH2, from the seeds of *V. hispanica* (Caryophyllaceae). Vaccatides contain 40 to 41 aa, including eight cysteine, eight glycine, and six glutamine residues. Transcriptomic analysis showed that vaccatides have a three-domain arrangement with a short C-terminal extender. Clustering analysis revealed that vaccatides may have originated differently from other known 8C-hevein-like peptides, which contain a short glycine- or alanine-rich remnant peptide at the C-terminal domain. Together, our results suggest that vaccatides represent a new hevein-like peptide subfamily, which has glutamine-rich mature and C-terminal domains.

## Materials and Methods

### Isolation and Purification of Vaccatides

Dried *Vaccaria hispanica* seeds were acquired from a local herbal distributor (Hung Soon Medical Trading Ltd., Singapore) and authenticated using their macroscopic and microscopic characteristics as previously described (People’s Medical Publishing House, 2010; Wong et al., 2015a,b). A voucher specimen (TCM-SVH-20161010) was stored at the Nanyang Technological University Herbarium, School of Biological Sciences, Singapore.

Dried *V. hispanica* seeds (2 kg) were pulverized by a blender and soaked in 20 L of MilliQ water at 4°C for 12 h. To remove the plant material, the mixture was passed through three layers of cheesecloth (Wong et al., 2013, 2017). The filtrate was centrifuged at 4°C at 9000 rpm for 20 min. A C_18_ flash column was prepared by packing 500 g of C_18_ powder (Grace, MD, USA) in a Büchner funnel (250 mm × 22 mm). The column was percolated with water and the filtrate was loaded onto the column under vacuum. The desired peptide was eluted with 60 and 80% (v/v) of ethanol. The 60% fraction was loaded onto a strong cation exchange flash column with Sepharose Fast Flow SP (GE Healthcare Life Sciences, Little Chalfont, United Kingdom). The ion exchange flash column was percolated with 20 mM potassium dihydrogen phosphate buffer (pH 3.0). The desired peptide was eluted with 1 M sodium chloride and 20 mM potassium dihydrogen phosphate buffer (pH 3.0). Multiple rounds of preparative RP-HPLC were performed to purify the desired peptides. The purification was carried out on a Prominence system (Shimadzu, Kyoto, Japan) attached to an Aeris peptide XB-C_18_ column (particle size 5 μm, 250 mm × 22 mm; Phenomenex, CA, United States) at a flow rate of 5 mL/min (Wong et al., 2014, 2016).

### Sequence Determination of the Vaccatides

The primary sequences of the vaccatides were determined by MS/MS sequencing as described previously (Nguyen et al., 2012, 2013). Vaccatides (10 μg) were re-dissolved in 20 mM dithiothreitol (30 μL). The mixture was incubated for 1 h at 37°C. The reduced vaccatides were alkylated by mixing with iodoacetamide (200 mM) and incubated for 1 h at 37°C. The mixture was desalted by a C_18_ Zip-tip. The *S*-alkylated vaccatides were analyzed by Dionex UltiMate 3000 UHPLC system equipped to an Orbitrap Elite mass spectrometer (Thermo Fisher Scientific Inc., Bremen, Germany). The composition of mobile phase A and B were 0.1% FA in deionized water and 0.1% FA in 90% acetonitrile with 10% deionized water, respectively.

The mass spectra was acquired by LTQ Tune Plus software (Thermo Fisher Scientific, Bremen, Germany) using positive mode by alternating the Full FT-MS as previously described (Wong et al., 2016). LC-MS/MS data was analyzed by PEAKS studio version 7.5 (Ma et al., 2003).

### Chitin-binding Assay

To determine the ability of vaccatide to bind to chitin, 40 μM of purified and *S*-alkylated vH2 was added to chitin beads (60 μL) (New England BioLabs, Massachusetts, United Kingdom) in chitin-binding buffer and incubated for 4 h at room temperature (Kini et al., 2015). After incubation, the chitin beads were washed with chitin binding buffer to remove unbound compounds. The bound compounds were eluted by mixing 500 mM acetic acid (pH 3.0). The samples were profiled by RP-HPLC and MALDI-TOF MS (ABI 4800, Applied Biosystem, MA, United States).

### Stability Assays

The thermal, acidic, endopeptidase, and exopeptidase stability assays were performed as previously described (Wong et al., 2016). Trypsin and carboxypeptidase A were acquired from Sigma–Aldrich (MO, United States). Briefly, the purified vaccatide vH2 was incubated with or without enzyme at the optimal temperature and with a buffer solution as recommended by the manufacturer. At each specific time point, the sample was aliquoted and analyzed by an Aeris peptide XB-C_18_ column (particle size 3.6 μm, 100 mm × 2.7 mm; Phenomenex, CA, United States) attached to an Acquity ultra-performance liquid chromatography H-class system (Waters, Milford, Japan). The areas under the peaks before and after treatment were determined to evaluate the stability.

### NMR Structural Determination

Two-dimension TOCSY and NOESY were performed on a Bruker 800 MHz NMR spectrometer (Bruker, IL, United States). Vaccatide vH2 was dissolved in 5% D_2_O and 95% H_2_O (pH 3.5) to a final concentration of 1 mM in a cryogenic probe. The mixing times for 2D TOCSY and NOESY were set at 80 ms and 200 ms, respectively. The spectrum width and carrier frequency were set at 12 and 4.745 ppm, respectively, and were analyzed by NMRPipe software (Delaglio et al., 1995). The assignment of NOE cross-peaks were determined by Sparky software (Goddard and Kneller). The distance restraints were classified into three classes: (1) strong: 0 < d ≤ 1.8 Å; (2) medium: 1.8 < d ≤ 3.4 Å; (3) weak: 3.4 < d ≤ 5 Å. Based on the hydrogen/deuterium exchange experiment, eight hydrogen bonds were selected for structural calculation using CNSsolve 1.3 software (Brunger et al., 1998). The distance between HN and O, and N and O were defined as 2.2–0.6 and 3.3–0.8 Å, respectively. Dihedral angle restraints were derived from the ^3^J_HN-Hα_ coupling constant observed in 1D ^1^H NMR spectrum. The backbone Φ angle was considered between –120° to –160° if the coupling constant was larger than 8 Hz. The structure was verified using the PROCHECK program (Laskowski et al., 1996) and displayed by Chimera version 1.6.2 (Huang et al., 1996). The solution 3D NMR structure of vaccatide vH2 was deposited on Protein Data Bank with an accession number of 5XDI.

### Anti-fungal Assay

A radial disk diffusion assay was employed to evaluate the anti-fungal activities of vaccatide against the mycelium growth of *Alternaria alternate* (CICC 40292), *Curvularia lunata* (CICC 40301), *Fusarium oxysporum* (CICC 2532) and *Rhizoctonia solani* (CICC 40259) were purchased from the China Center of Industrial Culture Collection (Beijing, China). The fungal strains were grown on potato dextrose agar (Sigma-Aldrich, MO, United States) in 90 mm × 15 mm Petri dishes. A circular agar with fungal mycelia was obtained by piecing a hole in the confluence fungal plate using a 1 mL pipette tip. The circular agar was placed on the center of a new agar plate and incubated for 48–72 h at 25°C. The plate was allowed to grow until the diameter of a fungal colony reached 3 cm. Subsequently, four circular filter papers were positioned 1 cm away from the mycelia colony. Lyophilized vaccatide vH2 was re-dissolved in 20 μL of phosphate buffer solution with concentrations of 17.5, 35, and 70 μg/mL and dripped on top of the circular filter paper. Negative control experiment was performed by adding 20 μL of phosphate buffer solution. The plate was incubated at 25°C for 48–72 h until the mycelia reached the rim of the negative control’s filter paper disk.

A microbroth dilution antifungal assay was employed to determine the IC_50_ levels of vH2 against different fungal strains. Fungal spores were obtained from a confluent agar plate. The spores were suspended in half-strength potato dextrose broth. The spore solution (80 mL; 3000 spores/mL) was mixed with vaccatide vH2 (20 μL) at different concentrations in a 96-well microplate and incubated at 25°C for 24 h. Methanol (100 μL) was added to fix the fungi for 30 min at room temperature. The methanol was decanted and 1% methylene blue stain dissolved in borate acid buffer (0.01 M; pH 8.5) was added. The plate was incubated at room temperature for 30 min. Subsequently, the stain was removed and washed with MilliQ water. Hydrochloric acid (50 mM) in 50% ethanol was added to re-dissolve the stain and the plate was measured at an absorbance of 640 nm by an Infinite 200 PRO microplate reader (Tecan, Männedorf, Switzerland).

### Data Mining, Bioinformatics, and Statistical Analyses

The vaccatide encoded gene (accession number: JZ158858.1) was obtained from the NCBI GenBank and was translated using EMBOSS Transeq (McWilliam et al., 2013). ATG was defined as the start codon whereas TAA, TAG, and TGA were defined as the stop codon. SignalP 4.0 was used to determine the cleavage site of the signal peptide (Petersen et al., 2011). Primary aa sequences and precursor sequences were aligned using multiple sequence alignment algorithm MUSCLE (Edgar, 2004). Pairwise alignment was performed using EMBOSS Water (Li et al., 2015). Neighbor-joining clustering algorithm was used to constructed a phylogenetic tree with bootstrap test of 1000 replicates in MEGA7 (Kumar et al., 2016). The distances were computed using the Poisson correction method with all positions containing gaps and missing data were eliminated. The phylogenetic tree were displayed using iTOL v3 (Letunic and Bork, 2016).

The inhibition cure was fitted to a least square ordinary regression using variable slopes and logarithm transformed x-axis. The IC_50_ was defined as the concentration of vaccatide vH2 required to inhibit 50% of the fungal growth. All statistical analyses were performed in triplicate and expressed as mean ± standard error of the mean (SEM) using GraphPad Prism version 6.1 (GraphPad software, CA, United States).

## Results

### Identification and Purification of Vaccatides

**FIGURE 2:**
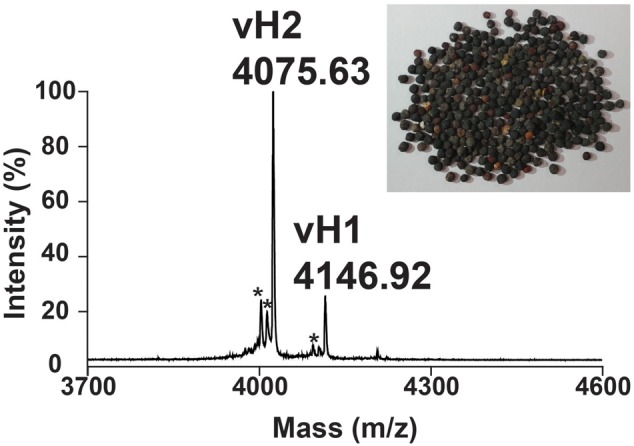
Mass spectrum of dried *Vaccaria hispanica* seeds. MALDI-TOF MS profile of the dried *V. hispanica* seeds extract in mass range of 3700–4600 Da. The dried *V. hispanica* seeds powder (1 g) was extracted with 5 mL of water. After centrifugation, the supernatant was fractionated using C_18_ Ziptip and eluted with 80% acetonitrile. ^∗^Unknown compounds. Further characterization is essential to determine the identifies of these compounds.

A mass spectrometry-guided approach was used to identify putative CRPs present in traditional Chinese medicine. Dried *V. hispanica* seeds (1 g) was extracted in boiling water (5 mL), fractionated by reversed-phase C18 chromatography and analyzed using MALDI-TOF MS. **Figure 2** shows a mass spectrum with a cluster of putative CRPs in the mass range of 4.0–4.5 kDa. To further characterize these peptides, a scale-up extraction was performed using 2 kg of dried *V. hispanica* seeds. Two compounds, designated as vaccatides vH1 and vH2 with relative monoisotopic molecular masses [M + H]^+^ of 4146.92 and 4075.63 Da, respectively, were isolated. The extraction yields of vH1 and vH2 were approximately 0.5 and 6.0 mg per kg of dried material, respectively.

A mass-shift experiment was performed to determine the number of cysteine residues present in these putative CRPs. Vaccatides vH1 and vH2 were reduced by dithiothreitol, *S*-alkylated by iodoacetamide and subsequently profiled using MALDI-TOF MS. The disulfide content was calculated as the mass difference before and after *S*-alkylation (Supplementary Figure S1, Supporting information). Since each *S*-alkylated cysteine residue increased in an additional mass of 58 Da, the vaccatides showed a mass shift of 464, suggesting that they contained eight cysteine residues.

### Primary Structure and Conserved Chitin-binding Domain of Vaccatides

**FIGURE 3:**
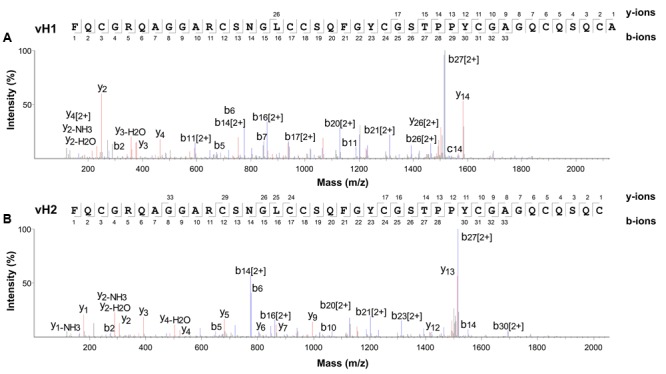
Mass spectra of vaccatides from LC-ESI-LTQ-Orbitrap MS/MS in positive ion mode. Vaccatide **(A)** vH1 and **(B)** vH2 were S-reduced by 20 mM dithiothreitol, *S*-alkylated with 200 mM iodoacetamide and subsequently desalted by C_18_ Ziptip. The spectrum was scanned between mass ranges of 100 and 1800 m/z. Assignment of isobaric aa (Lys/Gln and Ile/Leu) were confirmed by transcriptome data.

The aa sequences of vaccatides were determined by tandem mass spectrometry sequencing (**Figure 3**). The *S*-alkylated vaccatides were subjected to MALDI-TOF MS/MS, and their sequences were determined by comparing the mass differences between the *b*- and *y*-series ions. Susequently, the putative vaccatide vH1 sequence was deduced as F*X*CGR*X*AGGARCSNG*X*CCS*X*FGYCGSTPPYCGAG*X*C*X*S*X*CA, where *X* represents the isobaric residues Lys/Gln or Ile/Leu (**Figure 3A**). Their identities were confirmed from the Genbank cDNA sequence which gave a full sequence of FQCGRQAGGARCSNGL CCSQFGYCGSTPPYCGAGQCQSQCA. The difference between the calculated (4145.63 Da) and measured (4145.92 Da) molecular weight value was 0.29, suggesting other post-translational modifications were absent except for the formation of disulfide bonds. The primary sequences of vH2 were determined in the same manner (**Figure 3B**). Vaccatide vH2 is a truncated version of vH1 with a deletion of one aa residue (Ala41) at the C-terminus. Vaccatide vH2 was selected as the representative for further characterization because of its higher abundance than vH1. TBALSTN result revealed that vaccatides shared the identical type I chitin-binding with other reported 8C-hevein-like peptides and their sequences were summarized in **Table 1** (Broekaert et al., 1990), (Kini et al., 2017), (Fujimura et al., 2003), (Fujimura et al., 2005) and (Wong et al., 2016).

**Table 1:** Primary aa sequences and physiochemical properties of vaccatides and 8C-hevein-like peptides.

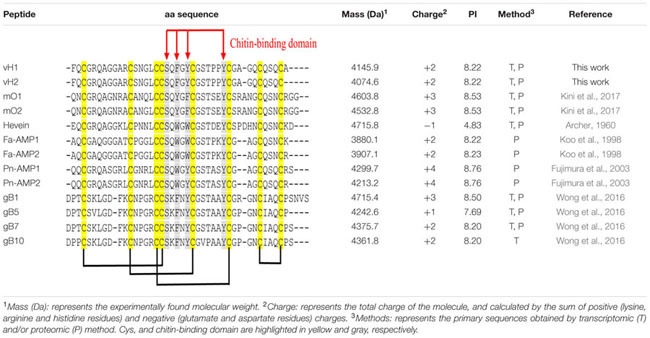

### Chitin-Binding Activity of Vaccatide

To examine the chitin-binding activity, the native and *S*-alkylated vH2 were employed (Supplementary Figure S2, Supporting information). After incubation with chitin beads, native vH2 was found in the acidic elution buffer but absent in the supernatant. In contrast, the *S*-alkylated and unfolded form of vH2 was found in the supernatant but absent in the eluent, suggesting the conformational importance of its chitin-binding domain.

### NMR Structure of Vaccatide

**FIGURE 4:**
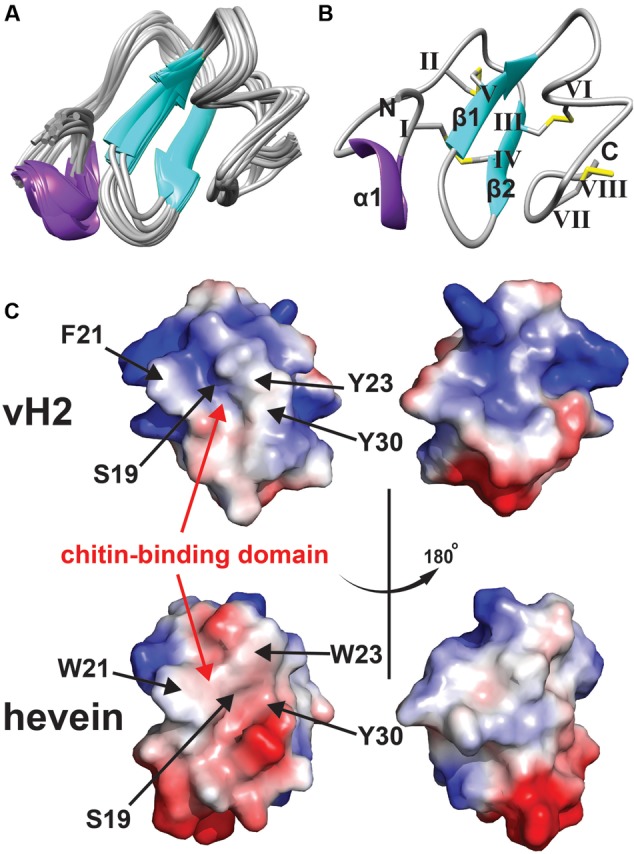
Solution NMR analysis of vH2. **(A)** Superposition of the vH2 backbone traces from the final 20 ensembles solution structures and restrained energy minimized structure. **(B)** Ribbon representation of vH2 structure solved by NMR. The disulfide connectivity is as follows: CysI–CysIV, CysII–CysV, CysIII–CysVI and CysVII–CysVIII. **(C)** Topology comparison of electrostatic surface between vH2 (PDB: 5XDI) and hevein (PDB: 1HEV) in two views revealing the chitin-binding domain. The distribution of electrostatic charges was displayed in red (negatively charged), blue (positively charged) and white (neutral). The residues (vH2: S19, F21, Y23, and Y30; hevein: S19, W21, W23, and Y30) within chitin-binding domain have been shown to play an important role in binding towards chitin.

The NMR solution structure of vH2 was obtained from the 2D ^1^H-^1^H-TOCSY and NOESY spectra illustrated in **Figure 4**. The sequential assignment was carried out based on the nuclear Overhauser (NOE) cross peaks between H*α*(*i*-1), H*β*(*i*-1), and HN(*i*). A total of 98% of proton resonance was assigned unambiguously. The vH2 solution structure was calculated from 434 distance restraints and 9 dihedral angle restraints (Supplementary Tables S1, S2, Supporting information). **Figure 4A** displays the 20 ensembles with a root-mean-square deviation value of 0.90 ± 0.23 Å for backbone atoms (Phe1 to Cys40) and 1.42 ± 0.27 Å for all heavy atoms. As illustrated in **Figure 4B**, vH2 comprised a well-defined structure with two short extended anti-parallel β-strands, where the β1 strand covers Cys17 to Ser19 and the β2 strand covers Tyr23 to Gly25, and a short one turn α-helix covers Arg5 to Ala7. The C-terminus of vH2 has no secondary structure (Ser26 to Cys40). The two proline residues at position 28 and 29 were arranged in the trans-conformation according to NOE cross peaks Hδ (Proi)–Hα (i-1).

The peptide vH2 contains four disulfide bonds, adopting the disulfide connectivity the same as the reported 8C-HLPs as: CysI–CysIV, CysII–CysV, CysIII–CysVI, CysVII–CysVIII. The disulfide bonds CysI–CysIV and CysII–CysIV connect the N-terminus towards the β1 and β2 sheets, respectively. The disulfide bond CysVII–CysVIII located at the extender alters the C-terminus to form a small loop between Glu37 and Glu39. The disulfide bond CysIII–CysVI links the C-terminus to the β2 sheet. The four disulfide bonds make the peptide structure well compacted, resulting in the high stability. As illustrated in **Figure 4C**, the surface charge of residues Ser19, Phe21, Tyr23, and Trp30 responsible for the chitin-binding property were neutral in vH2, whereas the two positively charged residues (Arg5 and Arg11) were distributed at the N-terminus opposite of the chitin-binding domain. In addition, no negatively charged residues were observed in vH2.

To evaluate whether vaccatide vH2 folded similarly to the reported hevein-like peptides and other knottins such as cystine knot α-amylase inhibitors, pairwise structural alignment algorithm TM-align was employed and displayed as the TM-score in **Table 2**. Vaccatide vH2 had a TM-score value of 0.509, 0.433, and 0.520 as compared to hevein, morintide mO1 and ginkgotide gB5, respectively. In contrast, the TM-score values of vH2 as compared to amaranthus α-amylase inhibitor AAI, allotide Ac4, and wrightide Wr-AI1 were 0.286, 0.241, and 0.299, respectively (Nguyen et al., 2014, 2015).

**Table 2:** TM align score between vaccatide vH2, 8C-hevein-like peptides, and cystine knot α-amylase inhibitors.

CRP family	Peptide	PDB	TM align score
8C-Hevein-like peptide	Hevein	1Q9B	0.50891
	gB5	N.A.	0.43324
	mO1	5WUZ	0.51973
Cystine knot α-amylase inhibitors	AAI	1QFD	0.28588
	Ac4	2MI9	0.24147
	Wr-AI1	2MAU	0.29919

*Pairwise alignment using vaccatide vH2 was performed. AAI, amaranthus α-amylase inhibitor; N.A., Not available. The structure of gB5 was kindly provided by the authors (Wong et al., 2016). A TM-score value <0.3 means that the two candidates have random structural similarity, while a value >0.5 means that they shared the same protein fold.*

### Stability of Vaccatide

**FIGURE 5:**
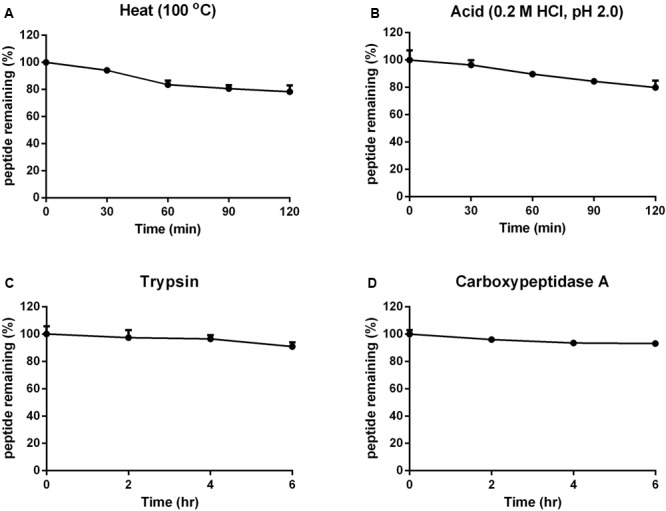
Stability of vH2 against various stimulated conditions. **(A)** Thermal stability of vH2 incubated at 100°C for 1 h. **(B)** Acidic stability of vH2 incubated in 0.2M HCl (pH 2.0) for 1 h. **(C)** Trypsin and **(D)** carboxypeptidase A enzymatic stability of vH2 incubated at 37°C for 6 h in buffer as suggested by the manufacturer. The experiment was performed in triplicate (*n* = 3) and expressed as mean ± standard error of the mean (SEM).

The stabilities of vaccatide vH2 against thermal, acidic and enzymatic denaturation were monitored by RP-HPLC and MALDI-TOF MS. **Figure 5** shows that vH2 displays a high tolerance to treatment with boiling water at 100°C or acidic pH 2 solution for 1 hr, with 78.2 and 79.8% of the peptide remaining, respectively. Additionally, vH2 was resistant to trypsin or carboxypeptidase A after 6 h incubation with >90% peptide remained.

### Anti-fungal Activity of Vaccatide

**FIGURE 6:**
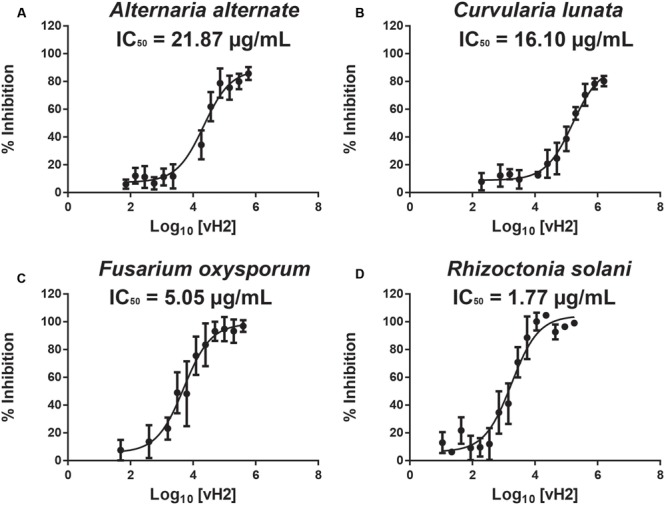
Microbroth dilution anti-fungal assay. Dose-response curves illustrate the inhibitory effect of vH2 on the fungal strains **(A)** *Alternaria alternate*, **(B)** *Curvularia lunata*, **(C)** *Fusarium oxysporum*, and **(D)** *Rhizoctonia solani*. The experiment was performed in triplicate (*n* = 3) and inhibition cure was fitted to a least square ordinary regression using variable slopes and logarithm transformed x-axis. The error bar was expressed as mean ± standard error of the mean.

**FIGURE 7:**
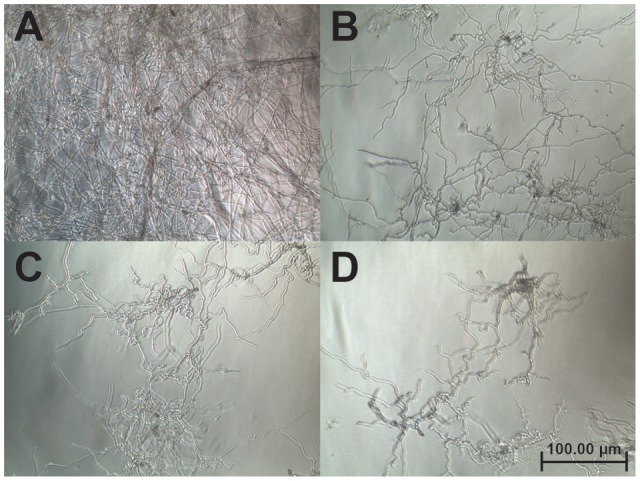
Bright-field microscopy of the mycelium growth of *Curvularia lunata* (100X). The anti-fungal assay was performed by the addition of **(A)** phosphate buffer saline as a control **(B)** 25, **(C)** 50, and **(D)** 100 μg/mL of vH2 in half-strength potato dextrose broth incubated at 25°C for 24 h.

A disk diffusion assay was used to evaluate the effects of vaccatide vH2 against mycelium growth of four common phyto-pathogenic fungal strains. The formation of crescent-shaped inhibition zones around vH2-treated disks indicated that *Alternaria alternate, Curvularia lunata, Fusarium oxysporum*, and *Rhizoctonia solani* were susceptible towards vH2 (Supplementary Figure S3, Supporting information). A microbroth dilution assay was subsequently used to determine the IC_50_ values against vH2-susceptible fungal strains. From the dose-response curves (**Figure 6**), the IC_50_ values against fungal strains *A. alternate, C. lunata, F. oxysporum* and *R. solani* are 21.8, 16.1, 5.0, and 1.7 μg/mL, respectively. Bright-field microscopy (**Figures 7B–D**) showed that the co-incubation of vH2 for 24 h at 25°C significantly modified the morphology of the fungal mycelium in a dose-dependent manner. It also resulted in shorter and highly branched hyphae, vacuolar granulation, and swollen hyphal tips as well as retarded conidial germination compared to the untreated control experiment (**Figure 7A**).

### Biosynthesis of Vaccatides

**FIGURE 8:**
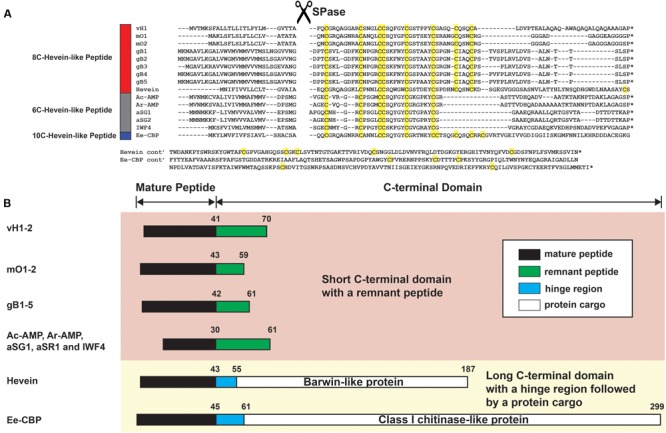
Gene alignment and biosynthetic pathway of hevein-like peptides. **(A)** Precursor sequences of vaccatides, hevein and other reported hevein-like peptides were aligned using MUSCLE. The precursors are divided into three major domains, including the signal peptide, mature peptide and C-terminal domain.^∗^Represents the stop codon. **(B)** Schematic diagram of the mature peptide and C-terminal domain. The C-terminal domain was divided into two forms: short and long. The short C-terminal domain contains a remnant peptide, whereas a hinge region followed by a protein cargo are present in the long C-terminal domain. The mature peptide, remnant peptide, hinge domain and protein cargo were filled with black, green, blue and white, respectively. The number displayed on top of each domain represents the average length of aa residues present. Genbank accession number: vH1 (JZ158858.1), mO1 (KY436355.1), mO2 (KY436356.1), gB1 (SRX087421), gB2 (EX935043.1), gB3 (CB075727.1), gB4 (CB094363.1) and gB5 (DR074391.1), hevein (AAO63572.1), Ac-AMP (CAA51210.1), Ar-AMP (AY861660.1), aSG1 (ALK00912.1), aSG2 (ALK00913.1), IWF4 (DN911508.1), and Ee-CBP (AAP35270.1).

The full-length vaccatide precursor sequence (JZ158858.1), designated as *vh1*, which encoded for vH1 and vH2, was obtained from the Genbank transcriptome database. **Figure 8A** compares the aligned precursor sequences of vaccatide and hevein-like peptides, including hevein (Broekaert et al., 1990), Ac-AMP from crop plant *Amaranthus caudatus* (De Bolle et al., 1996), IWF4 from beetroot *Bata vulgaris*, Ar-AMP from *Amaranthus retoflexus* (Lipkin et al., 2005), gB1–5 from *Ginkgo biloba* (Wong et al., 2016), aSG1 from green variety of *Alternanthera sessilis*, aSR1 from red variety of *Alternanthera sessilis* (Kini et al., 2015), mO1 from *Moringa oleifera* (Kini et al., 2017), and Ee-CBP from *Euonymus europaeus* (Van den Bergh et al., 2004). The vaccatide precursor sequence contain three domains, including an endoplasmic reticulum signal peptide which contains 25 aa, a mature domain (41 aa) and a short C-terminal domain (29 aa). A distinguishing feature of a vaccatide precursor sequence is the absence of a chimeric protein cargo at the C-terminal domain compared to the other 10C-hevein-like peptides and hevein, both of which carry an additional functional protein cargo.

## Discussion

### Occurrence of Hevein-Like Peptides *In Planta*

Hevein-like peptides were reported from both angiosperms and gymnosperms. Among angiosperms, a total of twelve 6C-hevein-like peptides were reported, and ten of which were isolated from the crop-plant Amaranthaceae family. For 8C- and 10C-hevein-like peptides, a total of 16 members were reported, and 12 of which were isolated from eudicots, with only four are found in monocots (Supplementary Table S3, Supporting information). Recently, our laboratory identified eleven proline-rich 8C-hevein-like peptides, ginkgotides, and an additional 85 ginkgotide-like peptides from 47 gymnosperms from transcriptomic data mining (Wong et al., 2016). The 8C- and 10C-hevein-like peptides are distributed in various families of angiosperms; however, all 6C-hevein-like peptides were isolated from the Caryophyllales order, including Ac-AMPs, Ar-AMP, aSGs, aSRs and IWF4 of the Amaranthaceae family (Broekaert et al., 1992; Nielsen et al., 1997; Lipkin et al., 2005) as well as SmAMPs from *Stellaria media* of the Caryophyllaceae family (Rogozhin et al., 2015). Apart from 6C-hevein-like peptides, the order of Caryophyllales contains two 8C-hevein-like peptides, Fa-AMP1 and Fa-AMP2 from the Polygonaceae family and none from the Caryophyllaceae family (Fujimura et al., 2003). The discovery of vaccatides vH1 and vH2 from Caryophyllaceae is the first report of 8C-hevein-like peptides from this family, suggesting that the occurrence of 6C- and 8C-hevein-like peptides is not restricted to certain plant families, and they are more diverse than previously thought.

### Sequence Comparison with Reported 8C-Hevein-Like Peptides

Pairwise sequence alignment demonstrated that vaccatides share a high sequence identity (57.6–76.9%) and similarity (69.7–79.5%) with hevein and reported hevein-like peptides, suggesting that vaccatides belong to the 8C-hevein-like peptide subfamily (Supplementary Table S4, Supporting information). Vaccatides vH1 and vH2 shared the least sequence similarity to ginkgotides. In general, sequence comparison of vaccatides with the reported 8C-hevein-like peptides from angiosperms revealed that 20 of 41 aa are absolutely conserved. The calculated charge of vH1 and vH2 was +2 at a pH of 7.0 with an isoelectric point of 8.22, which is similar to other reported 8C-hevein-like peptides (**Table 1**). Apart from the eight cysteine residues, intercysteinyl loops 3 and 4 of the chitin-binding domain are highly conserved in these chitin-binding peptides. This domain is highly conserved between vaccatides and reported 8C-hevein-like peptides with a S*X*Φ*X*Φ**CG*X*_4_Y motif, where *X* and *Φ* represent small and aromatic aa, respectively. Among vaccatides and the reported hevein-like peptides, serine and the third aromatic aa are conserved. However, the second and third aromatic aa are phenylalanine and tyrosine in vaccatides and ginkgotides, whereas tryptophan and tryptophan/phenylalanine are found in other 8C-hevein-like peptides. Together, these results suggest that vaccatides vH1 and vH2 share a higher similarity in sequence and biophysical properties with the 8C-hevein-like peptides from angiosperms than those from gymnosperms.

### Sequence Comparison with Cystine Knot α-Amylase Inhibitors

Plant CRPs are classified based on their sequence homology, cysteine spacing, disulfide connectivity and unique conserved domain. Vaccatides contain a conserved cysteine spacing of C–C–CC–C–C–C–C. The presence of consecutive cysteine residues (CC motif) is a unique feature in plant CRP families and the first six cysteine spacing, the C–C–CC–C–C motif, is shared by two plant subfamilies, cystine knot α-amylase inhibitors and 6C-hevein-like peptides (Nguyen et al., 2014, 2015).

Vaccatides and 6C-hevein-like peptides shared similar cysteine spacing and motif between loop 1 and loop 4, in which the fourth disulfide bond at the extender was absent in 6C-hevein-like peptides. Also, vaccatides comprise consecutive proline residues at positions 28 and 29 of loop 4, which is a characteristic feature in cystine knot α-amylase inhibitors such as allotides from *Allamanda cathartica* (Nguyen et al., 2015). However, cystine knot α-amylase inhibitors do not contain a chitin-binding domain. In addition, pairwise sequence alignment between vaccatide vH2 and cystine knot α-amylase inhibitors revealed that they share low sequence identity (25.9–40.0%) and similarity (33.3–50.0%). Although both families contain a cysteine knot core, cystine knot α-amylase inhibitors are significantly shorter in length (29–30 aa) and contain only six cysteine residues compared to the eight cysteine residues in vaccatides with an additional extender at the C-terminus. In addition, vaccatide vH2 does not display an observable inhibitory anti-α-amylase effect at concentrations up to 100 μM (Supplementary Figure S4, Supporting information). Together, our results suggested that vaccatides vH1 and vH2 are not cystine-knot α-amylase inhibitors.

### Highly Disulfide-Constrained Structure

The 8C-hevein-like peptide structures are characterized by two to three β-strands constrained by a cystine core at the N-terminus with an extra disulfide bond flanked at the extender of C-terminus (Andersen et al., 1993; Wong et al., 2016). By comparing the average energies of 15 different disulfide bond patterns, the disulfide connectivity of vH2 was identical to hevein obtained from X-ray crystallography (Rodriguez-Romero et al., 1991) and reported 8C-hevein-like peptides such as ginkgotide gB5 determined by solution NMR (Wong et al., 2016). Analysis of the surface topology revealed that most of the hydrophobic and eight cysteine residues were buried inside the cystine core (**Figure 4C**). Stability studies revealed that vH2 was highly stable against thermal, acidic and proteolytic degradation. These results agreed with previous reports of other CRPs, which have a high tolerance against various harsh conditions because of their disulfide-constrained compact structure (Nguyen et al., 2011a; Kini et al., 2015). The ability of vaccatide and other CRPs to withstand a simulated decoction preparation process and proteolytic denaturation reveals their relevance as potential active constituents in medicinal herbs and as scaffolds for peptide grafting.

A TM-score value of <0.3 represents random structural similarity between the two candidates, whereas a value of >0.5 represents that they shared the same protein fold (Zhang and Skolnick, 2005). Vaccatide vH2 has an average TM-score of 0.487 ± 0.038 and 0.276 ± 0.025 when compared to hevein-like peptides and cystine-knot α-amylase inhibitors, respectively (**Table 2**). Both hevein-like peptide and cystine-knot α-amylase inhibitors contain two anti-parallel β-sheets (Rodriguez-Romero et al., 1991; Nguyen et al., 2014, 2015; Wong et al., 2016). However, the first and second β-strands are located at the chitin-binding domain in hevein-like peptide, but are located near the C-terminus in cystine-knot α-amylase inhibitor. More importantly, the consecutive proline residues in vH2 are both arranged in a trans-conformation, whereas in allotide Ac4, the first and second proline residues were arranged in *cis*- and *trans*-conformations, respectively (Nguyen et al., 2015). Together, these results suggest that vH2 has similar protein folding to other members of 8C-hevein-like peptides but not cystine knot α-amylase inhibitors, although they share a similar cystine-knot core.

### Anti-fungal Activity

Vaccatide vH2 inhibits the mycelium growth of four fungal strains with IC_50_ values ranging between 1.77 and 21.87 μg/mL, which is comparable to other reported hevein-like peptides (Koo et al., 1998; Van den Bergh et al., 2002; Fujimura et al., 2003). Apart from mycelium growth inhibition, vH2 retards hyphae branching and promotes vacuolar granulation. These morphological modifications are similar to the previously reported experiments, including those using the 8C-hevein-like peptide ginkgotide gB5 from *Ginkgo biloba* (Wong et al., 2016) and Ee-CBP from *E. europaeus*, a 10C-hevein-like peptide (Van den Bergh et al., 2002).

Currently, the anti-fungal activities were supported by two different mechanisms. The first mechanism contributes to the ability to bind to chitin, which enables the hevein-like peptide to bind to newly formed chitin polymers in the hyphae tips. Subsequently, the peptide-polymer complex inhibits the formation of chitin nascent, disrupts the cell wall morphogenesis and hence retards fugal mycelium growth (Fujimura et al., 2003, 2005). The second mechanism relates to the small footprint and highly disulfide-constrained compact structure, which allows the hevein-like peptide to easily penetrate through the fungal cell wall, alter the fungal cell membrane polarity and lead to leakage of cytoplasmic materials (Huang et al., 2002; Lipkin et al., 2005; Odintsova et al., 2009). This proposed anti-fungal mechanism had been observed in other CRP families, including thionins (Florack and Stiekema, 1994) and plant defensins (Thevissen et al., 1996). However, a detailed mechanism of anti-fungal activity exerted by hevein-like peptides remains to be elucidated, and further experiments to clarify this issue are warranted.

### Glutamine-Rich 8C-Hevein-Like Peptides

Similar to other hevein-like peptides, vaccatide precursor *vh1* comprises a three-domain architecture (De Bolle et al., 1996; Lipkin et al., 2005; Kini et al., 2015). The presence of a signal peptide suggests that vaccatide is a secretory peptide similar to other hevein-like peptides (Kini et al., 2015). The release of a mature vaccatide involves the cleavage of a signal peptide by signal peptidase and the removal of the C-terminal domain by endopeptidase. Subsequently, the mature vaccatide was transported to the Golgi apparatus for post-translational modifications and packed into vesicles for secretion (Nguyen et al., 2014, 2015).

**Figure 8B** summarizes the arrangement of the mature peptide and C-terminal domains among hevein-like peptides. Based on the length of the C-terminal domain, hevein-like peptides were categorized into two classes: short and long. The short C-terminal domain contains a remnant peptide, whereas a hinge region followed by a protein cargo are present in the long C-terminal domain. Among hevein-like peptides with a short C-terminal domain, the average lengths of the remnant peptides were 29, 16, 19, and 31 aa in vaccatides, morintides, ginkgotides, and 6C-hevein-like peptides, respectively. In contrast, the lengths of long C-terminal domains varied significantly. The lengths of the hinge region and protein cargo in hevein and Ee-CBP were 12 and 132 and 16 and 238 aa, respectively. In hevein, the protein cargo was encoded for a barwin-like protein, while that in Ee-CBP was encoded for a class 1 chitinase-like protein.

**FIGURE 9:**
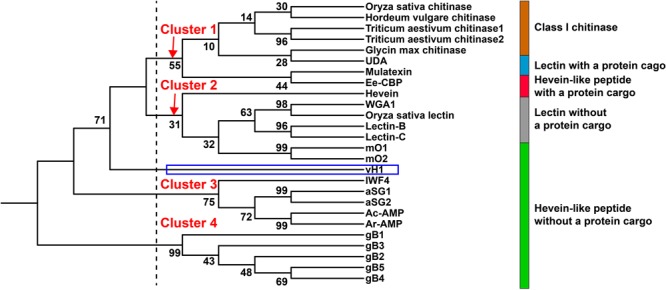
Phylogenetic tree of hevein-like peptides. Neighbor-joining clustering algorithm was employed to analyze the aligned precursor sequences and displayed as a phylogenetic tree using iTOL. Apart from the hevein-like peptides mentioned in **Figure 8**, the alignment includes class I chitinase such as *Triticum aestivum* chitinase 1 (AAR11388.1) and 2 (CAA53626.1) from *Triticum aestivum, Hordeum vulgare* chitinase (AAA56787.1) from *Hordeum vulgare, Oryza sativa* chitinase from *Oryza sativa* (AAA18585.1), *Glycin max* chitinase from *Glycin max* (AAF17593.1) and chitin-binding lectins such as WGA1 from *Triticum aestivum* (M25537.1), *Oryza sativa* lectin from *Oryza sativa* (AAA18585.1), lectin-B (BAB40792.1), and lectin-C (BAB21577.1) from *Phytolacca Americana*, UDA from *Urtica dioica* (M87302.1) and mulatexin from *Morus alba* (ABS86614.2).

**Figure 9** illustrates the relationship between vaccatides and proteins with a chitin-binding domain by a neighbor-joining clustering algorithm. Four major clusters, designated as Cluster 1 to 4, were observed. Cluster 1 belongs to class I chitinase, whereas Cluster 3 and 4 refer to 6C-hevein-like peptides and ginkgotides, respectively, both of which contain a remnant peptide instead of a long protein cargo at the C-terminus. Cluster 2 represents lectin without a protein cargo and is overlapped with hevein with and without a protein cargo. Interestingly, vaccatide precursor (boxed in blue) was an outlier and located between Cluster 2 and 3, suggesting that vaccatides represent a group of hevein-like peptides that are different to other reported hevein-like peptides and proteins containing one or multiple chitin-binding domain(s).

Apart from hevein, all reported 8C-hevein-like peptide precursors are protein-cargo-free (**Figure 8B**). Instead, they contain a remnant peptide with 16–34 aa at the C-terminal domain. In the vaccatide precursor sequence, there are six glutamine residues in the mature peptide, with six glutamine and 12 alanine residues in the remnant peptide, whereas morintide comprised a glycine-rich mature (six residues) and remnant peptides (eleven residues). In contrast, ginkgotides from gymnosperm, contain a proline-rich core-peptide (three to six residues) and a remnant peptide rich in serine and lysine residues (four and three residues, respectively). Although the remnant peptides differ among species, they all ended with a proline residue before the stop codon. Kini et al. (2017) speculated that the occurrence of a remnant peptide in the morintide precursor sequence could be a result of frame-shift deletions or mRNA splicing events. It is speculated that the morintide precursor originally consisted of a chitinase gene because the remnant peptide shared a similar architecture as the hinge region in chitinase, which is rich in glycine residues. The chitinase gene is either removed by a frame shift deletion of the chitinase domain DNA or by alternative splicing of chitinase pre-mRNA, followed by reverse transcription, and genomic integration (Andreev et al., 2012). However, this explanation cannot apply to vaccatides because the remnant peptide is rich in glutamine and alanine instead of glycine residues, suggesting that it may have originated from another source. This speculation is supported by the phylogenetic tree, in which vaccatide precursor is an outlier and does not belong to any major clusters. Together, it can be concluded that vaccatides are a new subfamily of 8C-hevein-like peptides, which contains glutamine-rich mature and remnant peptides.

## Conclusion

In this study, two glutamine-rich 8C-hevein-like peptides (vaccatides vH1 and vH2) were characterized from the dried *V. hispanica* seeds. Solution NMR studies showed that the vH2 structure is highly compact and disulfide-constrained, and it consists of four disulfide bonds, three arranged as a cystine knot and the fourth disulfide as an extender loop. The presence of multiple disulfide bonds renders vH2 a high tolerance to thermal, chemical and proteolytic denaturation, making it a potential scaffold for grafting metabolically stable and orally bioavailable peptidyl therapeutics. Transcriptomic data revealed that the vaccatide precursor sequence is protein-cargo-free, which differentiates it from the prototype hevein and the other protein-cargo-containing 10C-hevein-like peptides. Neighbor-joining clustering analysis showed that vaccatides are a new subfamily of 8C-hevein-like peptides, in which both the mature and C-terminal remnant peptides are glutamine-rich. Taken together, our results suggest that vaccatides may have originated differently than the other known 8C-hevein-like peptides, which contain a short glycine- and/or alanine-rich remnant peptide at the C-terminal domain. Our discovery reveals that hevein-like peptides are more structurally and biosynthetically diverse than we are anticipated.

## Author Contributions

JT, KW, and WT conceived and designed the experiments. KW, SK, WT, AS, and TX performed the experiments, analyzed the data, and wrote the manuscript. JT, SS revised the manuscript. All authors read and approved the final version of the manuscript.

## Conflict of Interest Statement

The authors declare that the research was conducted in the absence of any commercial or financial relationships that could be construed as a potential conflict of interest. The reviewer WP and handling Editor declared their shared affiliation, and the handling Editor states that the process met the standards of a fair and objective review.

## Abbreviations

aa: amino acids
CRPs: cysteine-rich peptides
FA: formic acid
IC_50_: half-maximal inhibitory concentration
MALDI-TOF MS: matrix-assisted laser desorption/ionization time-of-flight mass spectrometry
NCBI: National Center for Biotechnology Information
NOESY: nuclear Overhauser spectroscopy
RP-HPLC: reversed-phase high-performance liquid chromatography
TFA: trifluoroacetic acid
TOCSY: total correlation spectroscopy
UDA: *Urtica dioica* agglutinin
WGA: wheat germ agglutinin
