# Supplementary material for: Vaccatides: Antifungal Glutamine-Rich Hevein-Like Peptides from Vaccaria hispanica
Source: Front Plant Sci. 2017 Jun 21;8:1100. doi: 10.3389/fpls.2017.01100 (PMC5478723; doi:10.3389/fpls.2017.01100)
Supplement: Supplementary file 1 [file Table_1.DOCX]

Table S1. NMR experimental and structural statistics of vaccatide vH2.

| **NMR Distance Restraints** |  |
| --- | --- |
| Intra-Residue NOE (\|i-j\|=0) | 132 |
| Sequential NOE(\|i-j\|=1) | 124 |
| Medium-Range NOE (1<\|i-j\|≤5) | 72 |
| Long-Range NOE (\|i-j\|>5) | 106 |
| All | 434 |
| Hydrogen Bonds | 8 |
| Dihedral Angle Restraints | 9 |
| **Structural Statistics (36 residues, F1-C40)** |  |
| NOE Violation | 0.030 ± 0.001 Å |
| Maximum NOE Violation | 0.033 Å |
| Dihedral Angle Violation | 0.461 ± 0.088° |
| Maximum NOE Violation | 0.660° |
| Ramachandran Plot Region (36 residues) |  |
| Residues in Most Favored Regions | 16 (57.1%) |
| Residues in Additional Allowed Regions | 11 (39.3%) |
| Residues in Generously Allowed Regions | 1 (3.6%) |
| Residues in Disallowed Regions | 0 (0%) |
| Number of End-Residues (excl. Gly and Pro) | 2 |
| Number of Glycine Residues | 8 |
| Number of Proline Residues | 2 |
| **Mean RMSD from the Average Coordinates (40 residues, F1-C40)** | |
| Backbone Atoms | 0.90 ± 0.23 Å |
| Heavy Atoms | 1.42 ± 0.27 Å |

Experimental Restraints and Structural Statistics of 20 Lowest-Energy Structures of vH2 among the 100 Structures Generated by CNSsolve 1.3.
