# Supplementary material for: Vaccatides: Antifungal Glutamine-Rich Hevein-Like Peptides from Vaccaria hispanica
Source: Front Plant Sci. 2017 Jun 21;8:1100. doi: 10.3389/fpls.2017.01100 (PMC5478723; doi:10.3389/fpls.2017.01100)
Supplement: Supplementary file 2 [file Table_2.DOCX]

Table S2. NMR atom chemical shift list of gB5.

| **ResID** | **HN(ppm)** | **Hα (ppm)** | **Hβ (ppm)** | | **Others** |
| --- | --- | --- | --- | --- | --- |
| F1 |  | 3.505 | 2.644 | 3.198 | Hδ, 7.224 |
| Q2 | 8.535 | 4.495 | 2.129 | 2.019 | Qγ, 2.452 |
| C3 | 7.838 | 4.823 | 2.997 | 2.615 |  |
| G4 | 8.255 | 3.797, 3.659 |  |  |  |
| R5 | 9.105 | 3.973 | 1.776 | 1.696 | Hγ, 1.599, 1.429; Hδ, 3.136 |
| Q6 | 9.074 | 3.771 | 1.414 | 0.904 | Qγ, 1.936; Hδ, 7.627, 7.254 |
| A7 | 7.412 | 4.669 | 0.897 |  |  |
| G8 | 7.985 | 3.836 |  |  |  |
| G9 | 8.293 | 4.111, 3.3 |  |  |  |
| A10 | 6.93 | 4.155 | 1.445 |  |  |
| R11 | 8.548 | 4.488 | 1.906 | 1.715 | Hγ, 1.936, 1.444 |
| C12 | 9.565 | 4.648 | 3.049 | 2.595 |  |
| S13 | 8.86 | 4.386 | 3.902 | 3.819 |  |
| N14 | 8.582 | 4.318 | 2.839 | 2.724 |  |
| G15 | 8.699 | 4.137, 3.621 |  |  |  |
| L16 | 7.566 | 4.381 | 1.838 | 1.496 | Hγ, 1.315; Hδ, 1.286, 0.76 |
| C17 | 8.717 | 4.937 | 4.165 | 2.505 |  |
| C18 | 8.238 | 5.266 | 3.244 | 2.828 |  |
| S19 | 10.129 | 5.302 | 4.58 | 4.404 |  |
| Q20 | 8.962 | 4.094 | 1.739 |  | Hγ, 1.900, 1.505; Hδ, 6.981, 6.834 |
| F21 | 7.492 | 4.699 | 3.044 | 3.744 | Hδ, 6.493, 6.179 |
| G22 | 7.871 | 3.963, 3.621 |  |  |  |
| Y23 | 7.507 | 5.071 | 2.95 | 3.562 | Hδ, 7.161, 6.696 |
| C24 | 8.681 | 5.933 | 2.833 | 2.84 |  |
| G25 | 9.085 | 3.675, 2.115 |  |  |  |
| S26 | 8.823 | 5.202 | 4.119 | 3.671 |  |
| T27 | 7.332 | 4.805 |  |  | Hγ, 1.356; |
| P28 |  | 4.351 | 2.233 |  | Hγ, 2.013, 1.902; Hδ, 3.767, 3.695 |
| P29 |  | 4.416 | 2.444 | 2.266 | Hγ, 2.074, 2.006; Qδ, 3.999 |
| Y30 | 8.135 | 4.178 | 2.957 | 2.906 | Hδ, 7.380 |
| C31 | 7.404 | 4.721 | 3.304 | 2.514 |  |
| G32 | 9.097 | 3.98, 3.829 |  |  |  |
| A33 | 8.504 | 4.217 | 1.409 |  |  |
| G34 | 8.87 | 4.226, 3.744 |  |  |  |
| Q35 | 7.973 | 4.365 | 2.06 | 1.646 | HΥ, 2.377, 2.253; |
| C36 | 7.472 |  | 3.432 | 2.722 |  |
| Q37 | 9.853 | 4.686 | 1.762 |  | HΥ, 2.172, 2.088; Hδ, 8.046, 8.372 |
| S38 | 7.98 | 4.484 | 4.16 | 3.908 |  |
| Q39 | 8.909 | 3.823 | 2.26 | 2.168 | Hγ, 2.468, 2.403; Hδ, 7.393, 7.826 |
| C40 | 7.845 | 4.837 | 3.576 | 2.719 | Hδ, 7.224 |
