# Supplementary material for: Vaccatides: Antifungal Glutamine-Rich Hevein-Like Peptides from Vaccaria hispanica
Source: Front Plant Sci. 2017 Jun 21;8:1100. doi: 10.3389/fpls.2017.01100 (PMC5478723; doi:10.3389/fpls.2017.01100)
Supplement: Supplementary file 3 [file Table_3.DOCX]

Table S3. The scientific, clade, order and family of hevein-like peptides.

| Peptide | Scientific name | Clade | Order | Family |
| --- | --- | --- | --- | --- |
| 6C-Hevein-like peptide | |  |  |  |
| Ac-AMP1 and Ac-AMP2 | *Amaranthus caudatus* | Eudicots | Caryophyllales | Amaranthaceae |
| Ar-AMP | *Amaranthus retroflexus* | Eudicots | Caryophyllales | Amaranthaceae |
| IWF4 | *Beta vulgaris* | Eudicots | Caryophyllales | Amaranthaceae |
| aSG1‒3 and aSR1‒3 | *Alternanthera sessilis* | Eudicots | Caryophyllales | Amaranthaceae |
| SmAMP1 and SmAMP3 | *Stellaria media* | Eudicots | Caryophyllales | Caryophyllaceae |
| 8C-Hevein-like peptide | |  |  |  |
| Hevein | *Hevea brasiliensis* | Eudicots | Malpighiales | Euphorbiaceae |
| Fa-AMP1 and Fa-AMP2 | *Fagopyrum esculentum* | Eudicots | Caryophyllales | Polygonaceae |
| Pn-AMP1 and Pn-AMP1 | *Ipomoea nil* | Eudicots | Solanales | Convolvulaceae |
| mO1 and mO2 | *Moringa olerifera* | Eudicots | Brassicales | Moringaceae |
| vH1 and vH2 | *Vaccaria hispanica* | Eudicots | Caryophyllales | Caryophyllaceae |
| gB1‒11 | *Ginkgo biloba* | Ginkgophyta | Ginkgoales | Ginkgoaceae |
| 10C-Hevein-like peptide | |  |  |  |
| EAFP1 and EAFP2 | *Eucommia ulmoides* | Eudicots | Garryales | Eucommiaceae |
| Ee-CBP | *Euonymus europaeus* | Eudicots | Celastrales | Celastraceae |
| WAMP1‒4 | *Triticum kiharae* | Monocots | Poales | Poaceae |
