# Supplementary material for: Vaccatides: Antifungal Glutamine-Rich Hevein-Like Peptides from Vaccaria hispanica
Source: Front Plant Sci. 2017 Jun 21;8:1100. doi: 10.3389/fpls.2017.01100 (PMC5478723; doi:10.3389/fpls.2017.01100)
Supplement: Supplementary file 4 [file Table_4.DOCX]

Table S4. Pairwise alignment of vaccatides with 8C-hevein-like peptides and cystine-knot α-amylase inhibitors.

| **Peptide** | **Identity** | **Similarity** | **Gap** |
| --- | --- | --- | --- |
| **8C-Hevein-like peptides** | |  |  |
| Hevein | 65.0 | 70.0 | 2.5 |
| Fa-AMP1 | 74.4 | 79.5 | 2.6 |
| Fa-AMP2 | 74.4 | 79.5 | 2.6 |
| Pn-AMP1 | 76.9 | 79.5 | 2.6 |
| Pn-AMP2 | 76.9 | 79.5 | 2.6 |
| mO1 | 64.1 | 71.8 | 2.6 |
| mO2 | 66.7 | 74.4 | 2.6 |
| gB1 | 57.6 | 69.7 | 0 |
| gB5 | 57.6 | 69.7 | 0 |
| gB7 | 57.6 | 69.7 | 0 |
| gB10 | 57.6 | 69.7 | 0 |
| **Cystine-knot α-amylase inhibitors** | |  |  |
| Ac1 | 25.9 | 33.3 | 40.7 |
| Ac2 | 38.5 | 50 | 19.2 |
| Ac3 | 38.5 | 50 | 19.2 |
| Ac4 | 25.9 | 33.3 | 40.7 |
| Ac5 | 29.6 | 33.3 | 40.7 |
| As1 | 38.5 | 50 | 19.2 |
| As2 | 38.5 | 46.2 | 19.2 |
| As3 | 29.6 | 33.3 | 40.7 |
| As4 | 31.0 | 34.5 | 31 |
| Wr-AI1 | 37.1 | 48.6 | 20 |
| Wr-AI2 | 40.0 | 45.0 | 5 |
| Wr-AI3 | 37.1 | 48.6 | 20 |
| AAI | 28.0 | 44.0 | 8 |
