# Supplementary material for: Vaccatides: Antifungal Glutamine-Rich Hevein-Like Peptides from Vaccaria hispanica
Source: Front Plant Sci. 2017 Jun 21;8:1100. doi: 10.3389/fpls.2017.01100 (PMC5478723; doi:10.3389/fpls.2017.01100)
Supplement: Supplementary file 5 [file Data_Sheet_1.DOCX]

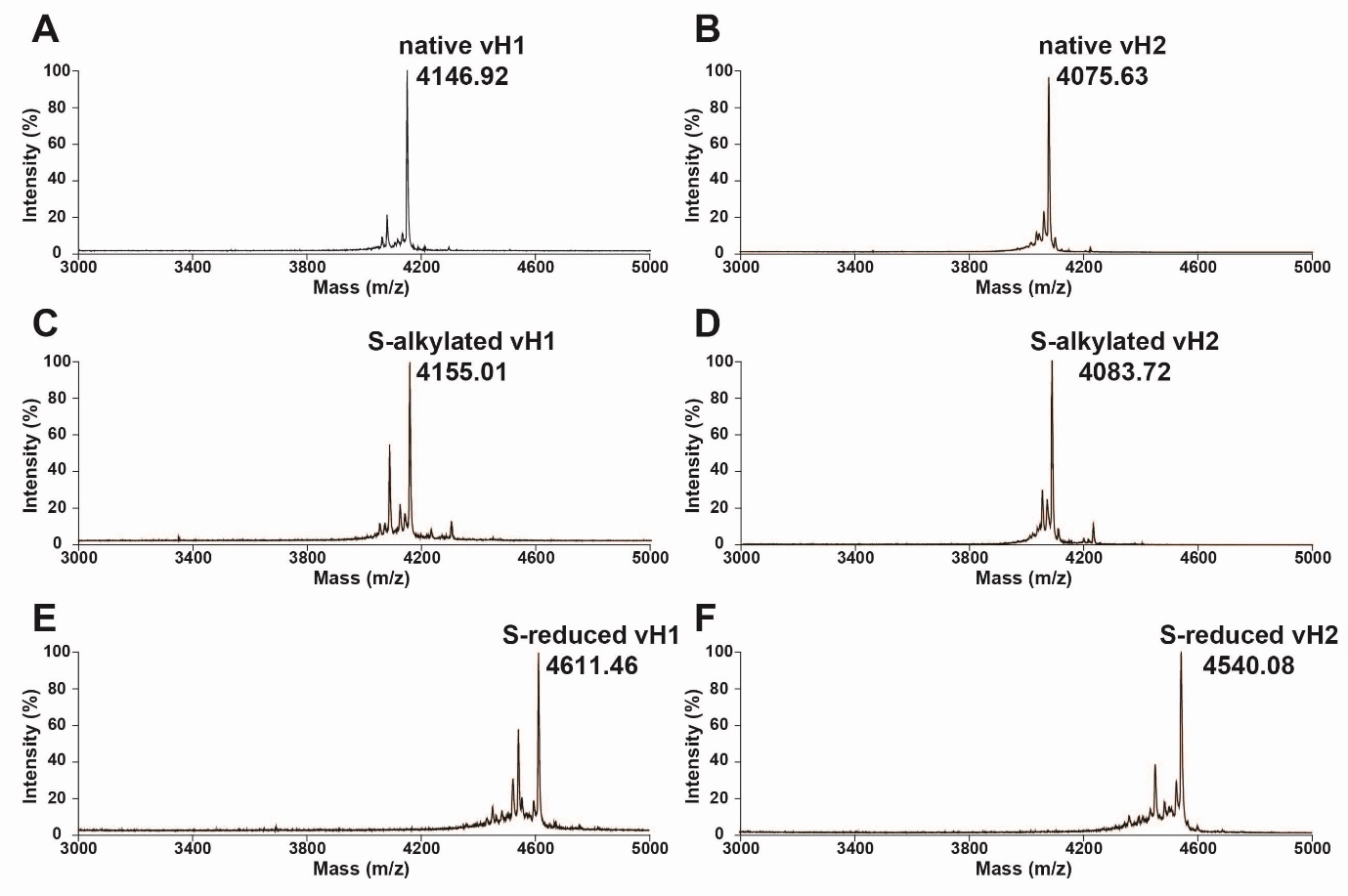


Figure S1. The MALDI-TOF spectra of vaccatides. The native vaccatides vH1 and vH2 (A & B) were S-reduced by dithiothreitol (C & D) and S-alkylated by iodoacetamide. The mass difference before and after the reductive S-alkylation of vaccatides vH1 and vH2 were monitored using MALDI-TOF MS. Each S-alkylated Cys caused a mass increase of 58 Da. A mass shift of 464 Da (E & F) suggests the presence of eight Cys in each peptide.
