# Supplementary material for: Vaccatides: Antifungal Glutamine-Rich Hevein-Like Peptides from Vaccaria hispanica
Source: Front Plant Sci. 2017 Jun 21;8:1100. doi: 10.3389/fpls.2017.01100 (PMC5478723; doi:10.3389/fpls.2017.01100)
Supplement: Supplementary file 6 [file Data_Sheet_2.DOCX]

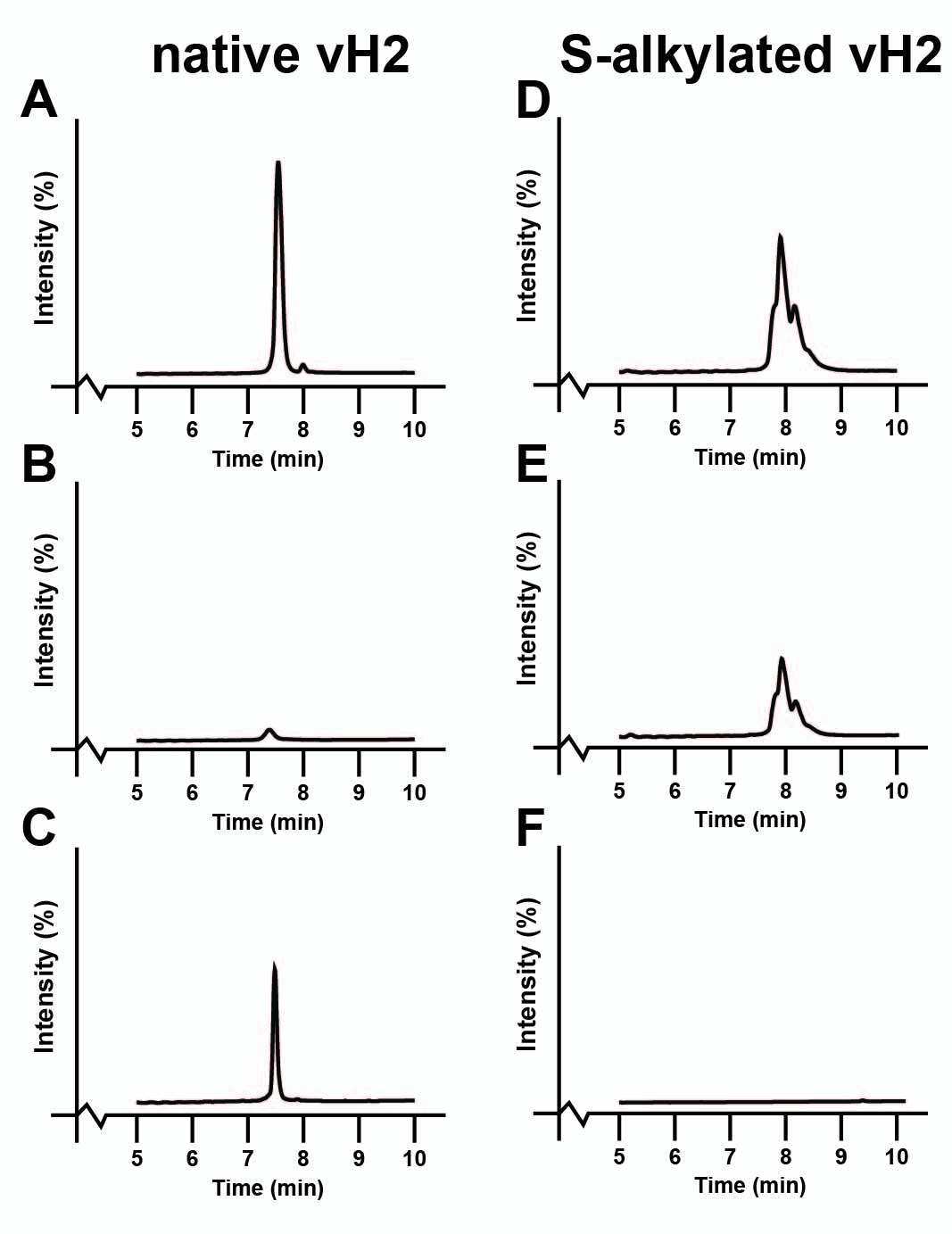


Figure S2. Chitin-binding activity of native and S-alkylated vaccatide. The native (A) and S-alkylated (D) vH2 was incubated with chitin beads in buffer (140 mM NaCl, 10 mM Tris, 1 mM EDTA and 0.1% (v/v) Tween at pH 8.0) for 4 hr. Chitin-bound peptides were eluted by 500 mM acetic acid (pH 3.0). The supernatants and eluents were analyzed using UPLC and MALDI-TOF MS.
