# Supplementary material for: Vaccatides: Antifungal Glutamine-Rich Hevein-Like Peptides from Vaccaria hispanica
Source: Front Plant Sci. 2017 Jun 21;8:1100. doi: 10.3389/fpls.2017.01100 (PMC5478723; doi:10.3389/fpls.2017.01100)
Supplement: Supplementary file 7 [file Data_Sheet_3.DOCX]

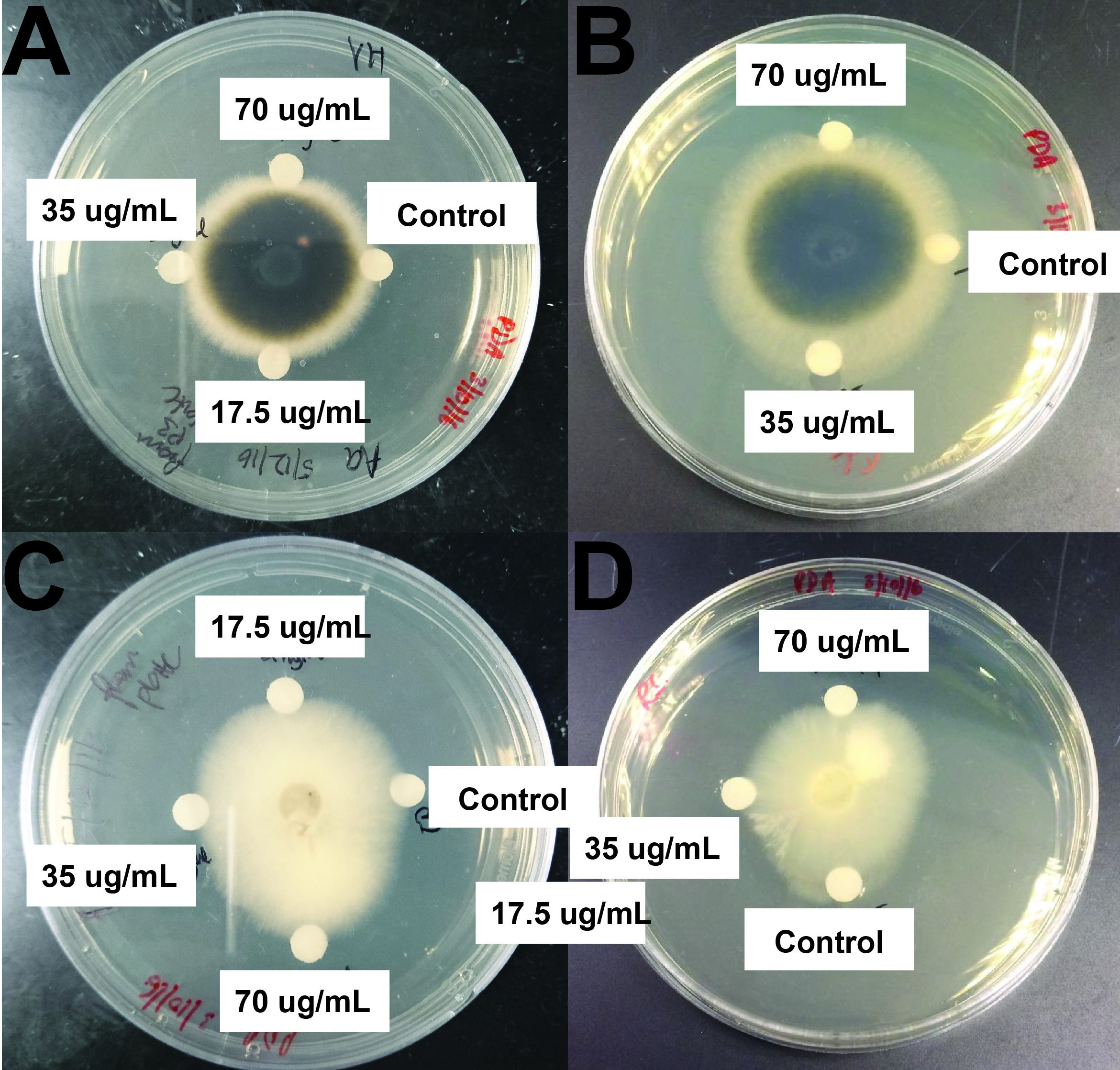


Figure S3. Anti-fungal disk diffusion assay of vH2. Crescent-shaped zones were observed around the vH2-treated disk with concentration of (2) 17.5, (3) 35 and (3) 70 µg/mL. (1) Milli-Q water was used as the control disk. These results suggested that vH2 exerted an anti-fungal activity against the hyphal growth of (A) *Alternaria alternate*, (B) *Curvularia lunata*, (C) *Fusarium oxysporum* and (D) *Rhizoctonia solani*.
