# Supplementary figures and images for: Vaccatides: Antifungal Glutamine-Rich Hevein-Like Peptides from Vaccaria hispanica
Source: Front Plant Sci. 2017 Jun 21;8:1100. doi: 10.3389/fpls.2017.01100 (PMC5478723; doi:10.3389/fpls.2017.01100)

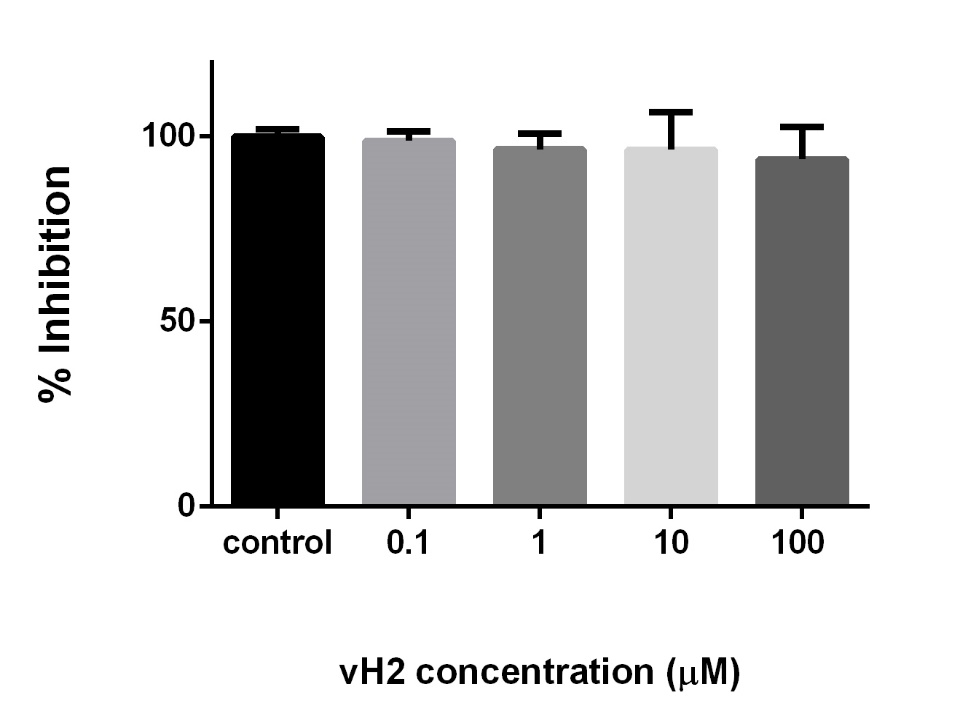


Figure S4. The effect of vaccatide vH2 on the *Tenebrio molitor* α-amylase activity.

Supplement: Supplementary file 8 [file Data_Sheet_4.DOCX]
